# Life after medicalised conception: an interpretative phenomenological analysis study exploring the experiences of pregnancy and transition to parenthood

**DOI:** 10.1186/s12884-025-07226-7

**Published:** 2025-02-11

**Authors:** Z. Foyston, L. E. Higgins, D. M. Smith, A Wittkowski

**Affiliations:** 1 Division of Psychology and Mental Health, School of Health Sciences, Faculty of Biology, Medicine and Health, The Manchester Academic Health Science Centre, The University of Manchester, Zochonis Building, Room 4.2, Brunswick Street, Manchester, M13 9PL UK; 2 The Perinatal Mental Health and Parenting (PRIME) Research Unit, Greater Manchester Mental Health NHS Foundation Trust, Manchester, UK; 3 Maternal and Fetal Health Research Centre, University of Manchester, Manchester, UK; 4 St. Mary’s Hospital, Manchester University Hospital NHS Foundation Trust, Manchester, UK; 5 Manchester Academic Health Science Centre, Manchester, UK

**Keywords:** Women, Parents, IVF, Antenatal, Postnatal, Qualitative, Lived experience

## Abstract

**Background:**

Pregnancy resulting from Medicalised Conception (MAC) is increasingly prevalent. In-Vitro Fertilisation (IVF) is the most common type of treatment which has been linked to increased pregnancy-specific anxiety and different approaches to parenting. This study explored the experiences of pregnancy and the transition to parenthood in individuals who conceived via IVF, identifying how they coped with any psychological difficulties.

**Method:**

Participants who successfully achieved pregnancy via IVF and had given birth to an infant aged 12 weeks to two years old, were interviewed. Interviews were audio-recorded, transcribed and analysed using Interpretative Phenomenological Analysis.

**Results:**

Based on data from 12 British participants, three superordinate themes were identified: (1) *The lasting imprint of IVF: unidentified and unmet psychological needs*. The resultant loss, grief and powerlessness associated with the IVF treatment left individuals emotionally vulnerable entering pregnancy. The lasting impact of IVF was evident, influencing birth preferences and feeding choices. (2) *The fragility of pregnancy: helpless and existing in a world of uncertainty*. Pregnancy was often approached with caution and trepidation, leading to methods of self-protection, such as difficulties in believing the existence of the pregnancy. (3) *The parental function of healthcare systems: needing an anchor and a sense of safety* highlighted the pivotal role of health systems and their ability to perpetuate or alleviate distress.

**Conclusions:**

The psychological vulnerability of parents after IVF needs to be considered throughout the perinatal period. Monitoring of psychological well-being and the implementation of specialist services and peer support for individuals who conceive via IVF are recommended.

**Supplementary Information:**

The online version contains supplementary material available at 10.1186/s12884-025-07226-7.

## Background

Medically Assisted Conception (MAC) has significantly increased over time [1–3]. In 2022, 2.9% of births in the United Kingdom (UK) resulted from in vitro fertilisation (IVF), the most commonly performed form of MAC (93% of UK MAC cycles) [4, 5]. In the UK, provision of assisted reproductive technologies, such as IVF, are highly regulated under the Human Fertilisation and Embryology Act [6]. Access to publicly funded IVF treatment is inconsistent across the UK, but access is almost exclusively restricted to childless couples [7]. Self-funded IVF treatment, through networks of private clinics and within NHS hospitals, account for 73% of all UK IVF treatments. Whilst infertility is the dominant indication for MAC (~ 90%), use of MAC for alternative reasons is rising [8]. Family structures are changing with an increase in same-sex couples and solo individuals pursuing MAC [9]. 

IVF treatment is physically and emotionally demanding, with effects persisting into the antenatal and postnatal period [10–12]. Pregnancy following MAC has unique physical [13–15] and psychological challenges [16]. In their 28-paper-review, Hammarberg et al. [11] consistently noted higher pregnancy-specific anxiety levels in individuals who became pregnant following MAC, compared to spontaneously conceiving individuals. Qualitative literature outlines similar findings. Individuals report difficulty in adjusting to pregnancy [17, 18], describing pregnancy as an emotionally turbulent time [19, 20]. Research into parental adjustment following MAC is inconclusive. Quantitative studies indicate psychological complexity [21], linked to lower self-esteem and parental confidence [16]. Qualitative studies suggest different parental experiences, including higher stress tolerance [22], and emotional investment in their child [23], leading to protective and permissive parenting styles [23, 24]. 

Current UK National Institute for Clinical Excellence guidance [25–27] does not list MAC as a risk factor for adverse perinatal mental health, offering no specific recommendation for subsequent care. There is no (inter)national healthcare professional guidance for pregnancy after MAC. Individuals perceive their antenatal and postnatal care as reductive and unattuned to their care needs [17, 19]. 

There is a growing body of qualitative literature exploring the parental voice following MAC internationally. However, most research is framed within the subfertility context, impairing understanding of whether such experiences following MAC result from the treatment itself or an unresolved subfertility issue [20]. It is imperative to understand the common experiences and needs of all parents who conceive through MAC, regardless of indication, to isolate the commonly experienced and specific effects of MAC from any preceding experience of subfertility, which has been the focus of the majority of the literature in this field to date. A meta-synthesis published by Rene et al. in 2022 [28]. and our own meta-synthesis [29] of 20 studies published in 2023 reflect the modern medical and social landscape of UK IVF services, which remain framed through the lens of subfertility. Given the rise of non-fertility related indications for MAC use, there is a need for further research with mixed samples or those who sought MAC for alternate reasons, focusing on the unifying experience of the medicalisation of conception and its effects on individuals’ experiences of pregnancy and the transition to parenthood. Furthermore, despite acknowledging increased psychological complexity of pregnancy after MAC, there is a qualitative research gap exploring how parents cope with reported difficulties. Coping mediates psychological distress, linked to adjustment levels [30, 31].

The first 1001 days, the period from conception to age two years, is a critical time for the emotional and physical development of the child [32]. Maternal psychological distress during the first 1001 days can affect mother-child bonding and early child emotional development. Therefore, this current study aimed to address these knowledge gaps by exploring (a) the lived experiences of pregnancy, birth and transition to parenthood following IVF-assisted conception for a range of indications and (b) how parents coped with any self-reported associated difficulties.

## Methods

### Design and ethical information

This qualitative study used Interpretative Phenomenological Analysis (IPA) [33, 34] to develop an in-depth understanding of the lived experiences of a small participant group within a complex, emotional context [35]. IPA is useful when ideas regarding a concept (such as MAC for non-fertility reasons) are not currently understood well from a lived experience perspective. The current study aimed to explore the common lived experiences of individual parents during pregnancy after MAC (rather than the process of treatment itself or a prior fertility journey), thereby exploring how they made sense of their experience of pregnancy after MAC and identifying points of convergence and divergence [34]. 

The Greater Manchester West Research Ethics Committee and the Health Research Authority, UK, granted ethical approval (Ref: 20/NW/0458).

### Participants

Adults were eligible to participate if they had 1) conceived via IVF following any indication, including subfertility, same-sex couples, solo-parenting and avoiding blood-borne or genetic condition transmission, 2) given birth to a singleton, living baby without serious fetal abnormalities 12 weeks to two years ago (parental report), 3) received IVF treatment and subsequent pregnancy care in the UK (whether publicly or privately funded) and 4) they were fluent in English. Individuals who 1) conceived spontaneously or used other non-IVF MAC techniques including ovulation induction and artificial insemination, 2) were under 18 years old or 3) could not give informed consent were excluded. There was no restriction on prior childbearing or previous failed IVF treatment cycles.

### Recruitment

Research study-specific accounts were created on Facebook, Twitter and Instagram. UK group pages, healthcare providers and charities relevant to IVF were contacted or ‘tagged’ on social media and an electronic study poster was shared.

Interested individuals were asked to self-refer. As we sought to explore the lived experiences of pregnancy and the transition to parenthood following MAC, our sample did not have to be homogenous by sharing similar demographic characteristics. To ensure the sample represented the varying reasons individuals seek IVF treatment (not just subfertility), purposive selection of participants took place and that included contacting a diverse group of charities, organisations or social media group pages, including those aimed at same-sex parents or solo parents.

Interested participants were provided with the participant information sheet and consent form. After an initial screening, those eligible were asked to provide informed (audio-recorded) consent before arranging an individual interview. However, as we intended to recruit participants with different reasons for seeking IVF treatment, we decided to decline to interview participants if their reasons were already represented in favour of recruiting eligible participants with reasons or backgrounds we had not yet heard from. Participants were informed they had two weeks to withdraw their data before their interview data entered the pseudo-anonymised dataset. To contextualise experience, participants were asked to complete a background questionnaire, incorporating details pertaining to participants’ IVF history.

### Interview

An interview topic guide (see supplementary file 1), comprising open-ended questions and follow up prompts [36], was developed, piloted and refined in consultation with clinicians and an individual with relevant lived experience. The guide covered four main areas: early pregnancy, later pregnancy, childbirth and their transition to parenthood. Particular attention was paid to pauses/silences, tone, language and metaphor choice to understand conveyed meaning. By allowing space for silence, participants were enabled to make links with their experience, further driving the conversation. Interviews ended with a participant wellbeing debrief, after cessation of recording, which included reviewing the participants’ emotional state and if they required any further support. All participants were provided with a debrief sheet detailing support services. Interviews were audio-recorded onto a separate encrypted device folder and transcribed verbatim by the first author.

### Data analysis

IPA [33, 34, 37] ensured rich examination of participants’ personal lived experiences, while considering their social world and current context, in particular the context of the medicalised nature of their conception. Its theoretical underpinnings with phenomenology, hermeneutics and idiography allow for the identification of patterns and divergences, both within and across narratives, which aligned with the research aims. Although the study design intentionally recruited participants from diverse backgrounds and with different reasons, the unifying experience being studied was of their experience of MAC and how that MAC experience influenced their experiences of pregnancy and their transition to parenthood.

**Fig. 1:**
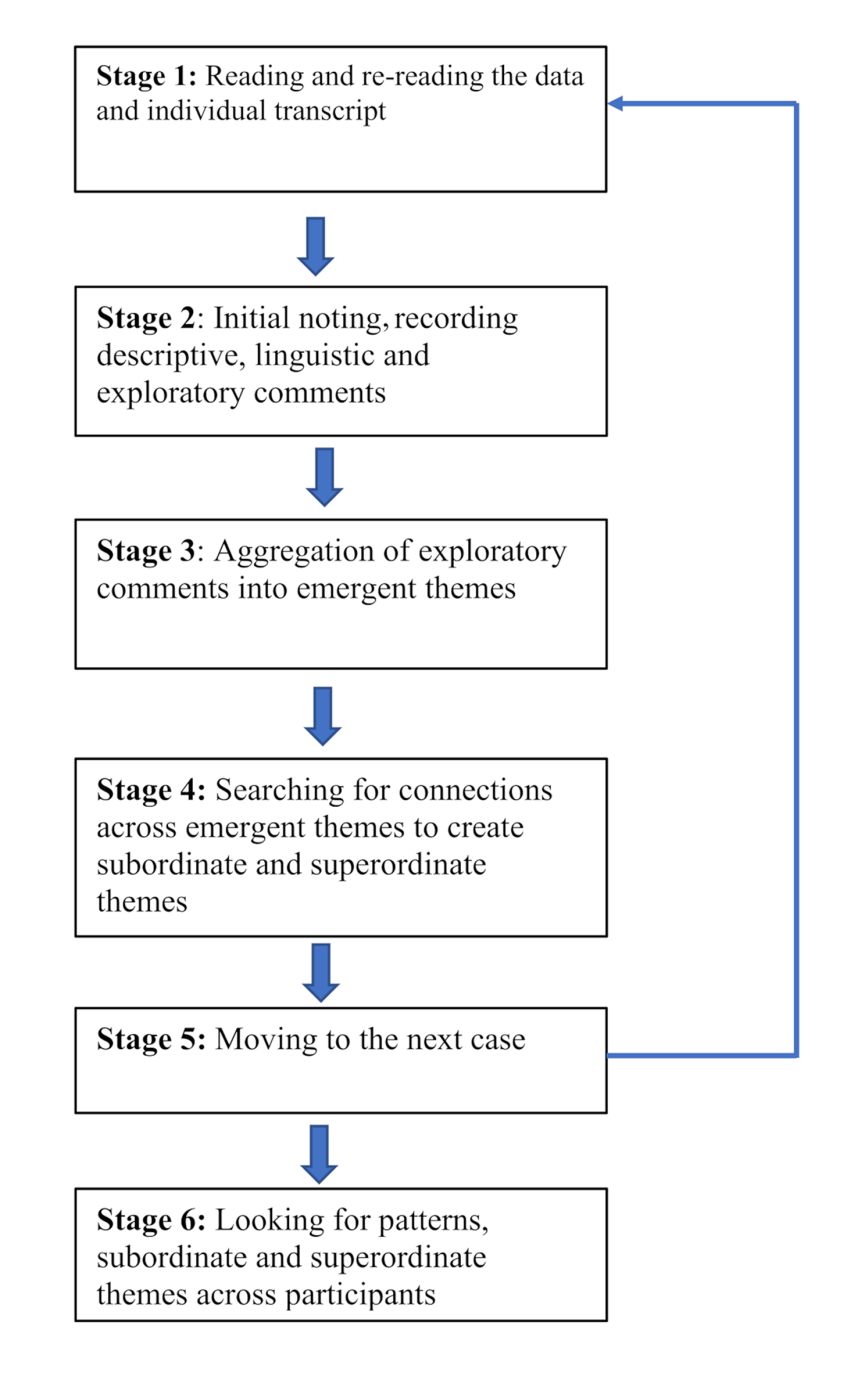
**Stages of IPA** adapted from Braun and Clarke (2006) [38]

During transcription, names were replaced with participants’ chosen pseudonym and all other identifiable information was removed. Transcript accuracy was checked against the audio-recording before the deletion of the recording. Transcripts were coded one at a time, line by line (see Fig. 1) [38]. Initial thoughts and ideas emerging from the data were noted and aggregated into emerging themes in Microsoft Word. Similarities, links and connections between emerging themes were identified and preliminary overarching superordinate themes assigned. Direct quotes to illustrate each theme were carefully chosen, protecting anonymity. The above process was then repeated for all other participants before superordinate themes across all participants were identified. A second researcher independently read each transcript and developed themes. The two researchers discussed emergent, subordinate and superordinate themes in relation to each participant’s story. All researchers reviewed the themes and agreed on them.

### Reflexivity and positioning statement

The double hermeneutic process in IPA means reflexivity is an essential component [39]. The research team comprised four white ciswomen, all professionally employed in healthcare and/or academia and who identified as mothers with varying reproductive experiences including IVF and perinatal loss. To account for the researchers’ pre-existing assumptions and beliefs, the first author kept a reflective journal to self-monitor biases and beliefs, reflecting on the intersecting relationships between themselves and the participants and considering both the insider and outsider positions [40] these afforded. These reflections were discussed in supervision, carefully considering how the main researcher’s own experiences might influence the findings. A critical realist position [41], which integrates realist ontology with a constructivist epistemology [42], was adopted.

## Results

### Sample characteristics

Twenty individuals expressed interest (18 postnatally, 2 antenatally) and received study information; five did not initiate further contact, one participant did not deliver before the end of data collection (02/12/2021). After screening revealed that two potentially eligible participants had very similar fertility challenges to those already recruited and interviewed, they were thanked for their interest and recruitment of participants was targeted towards same-sex couples or solo parents.

In total, 12 participants (11 cisgender women, 1 gender-nonbinary) were interviewed. The mean age was 37.5 years (range 31–43 years). Eleven were married or cohabiting and one was a solo parent. All held a university degree and identified as White British, living in the UK. Participants’ IVF indications included fertility challenges (*n* = 9), being in a female + non-binary couple (*n* = 2) or a solo parent (*n* = 1). Although one participant had given birth previously (stillborn), none had a prior living child and therefore none had prior experience of parenthood. Participants had undergone 1 to12 prior IVF treatment cycles, including the cycle leading to successful conception. Half of the cohort had experienced pregnancy loss including single (*n* = 1) or recurrent miscarriage (*n* = 4) and stillbirth (*n* = 1), while the other six individuals had no history of perinatal loss. All participants underwent IVF treatment between 2018 and 2020 in the UK. Table 1 outlines participants’ relevant characteristics. Interviews took place remotely via video conferencing software (*n* = 10) or telephone (*n* = 2), based on participant access/preference. Interviews ranged from 61 to148 (mean 94) minutes.

**Table 1:** Overview of some participant characteristics

Pseudonym	Gender	Reason for seeking IVF	Year of IVF treatment	Prior IVF treatment cycles	Pregnancy loss
Dee	Cisgender woman	Fertility challenges	2018	> 6	Recurrent miscarriage
Miriam	Cisgender woman	Fertility challenges	2020	2	Recurrent miscarriage
Jane	Cisgender woman	Fertility challenges	Not reported	> 6	No
Ever-hopeful	Cisgender woman	Fertility challenges	2018	3	Single miscarriage
Elle	Cisgender woman	Fertility challenges	2020	0	No
Gemma	Cisgender woman	Fertility challenges	2019	0	No
Abigail	Cisgender woman	Fertility challenges	2019	4	Stillbirth
Louise	Cisgender woman	Fertility challenges	2018	5	Recurrent miscarriage
Tel	Gender-nonbinary	Same-sex couple	2019	0	No
Kate	Cisgender woman	Solo parent	2019	0	No
Frankie	Cisgender woman	Fertility challenges	2020	0	Recurrent miscarriage
Sophie	Cisgender woman	Same-sex couple	2020	3	Chemical pregnancy; not considered a loss by the participant

*Note: To protect participant identity*,* IVF treatment cycles over 6 were truncated and their UK location was not reported*

### Findings

**Fig. 2:**
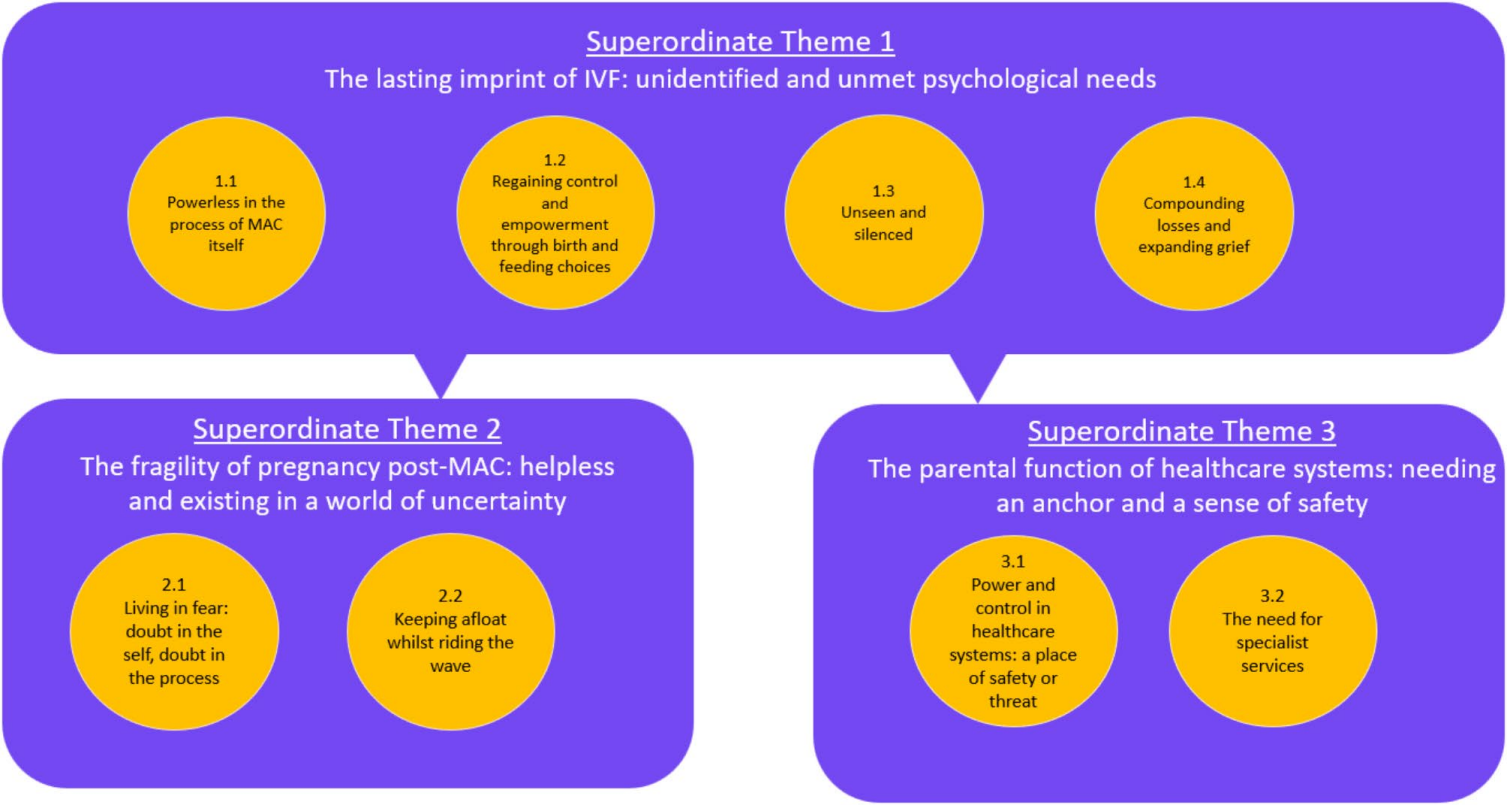
A conceptual map of superordinate and subordinate themes. Rectangular boxes represent superordinate themes; circle boxes represent subordinate theme

Three superordinate themes and nine subordinate themes were identified (see Fig. 2).

### Superordinate theme 1: the lasting imprint of IVF - unidentified and unmet psychological needs

The physical and emotional impact of the IVF process penetrated multiple aspects of participants’ lives, leaving unidentified and unmet needs. For many, IVF was a repeated, cumulative loss which impacted future experiences. This theme demonstrates IVF’s enduring impact from before conception through to parenting, encompassing four subordinate themes across this journey.

#### Subordinate theme 1.1: powerless in the process of MAC itself

Participants reflected on the uncertain and helpless position they found themselves in when they entered the IVF process. Louise described being at the “*mercy*” of IVF. The process was described as “*traumatic”* (*Dee*,* Tel*), *“stressful*” *(Abigail*,* Jane*,* Dee*,* Louise*,* Kate*,* Frankie)*, “*all-consuming*” (*Elle*,* Jane*,* Ever-Hopeful*,* Louise)*. IVF’s medical, intrusive and invasive nature was described as being associated with lacking bodily autonomy and control. For *Kate*, a solo parent, lacking participation in the process created doubt and anxiety that her baby was genetically related to her:

*“Not only do you not totally know that it’s his sperm*,* but you didn’t actually know it’s my egg… you just have that slight horror of what if there’s a mix up at the clinic….But*,* genuinely to start with*,* it did feel quite anxiety inducing that you didn’t know that she was…maybe that did affect why I didn’t have the rush of bonding immediately because I just didn’t know she was…”.*

Participants reported the treatment-associated obligations and highly controlled medical environment’s impact. The “*cold”* (*Tel)*, “*clinical” (Gemma*,* Tel*,* Kate)* nature of IVF contrasted with participants’ preconceived ideas, hopes and fantasies regarding conception. For some, like *Tel*, a gender-nonbinary individual in relationship with a ciswoman, the powerlessness experienced within the IVF process removed the nurturing aspect of conception and pregnancy leading to perceived negative impacts on bonding with the baby during pregnancy and parenting:

*“It was constantly this ‘go to the hospital*,* get checked’. It sort of removed the nurture from it*,* for me. It was all just so clinical and umm*,* cold…it certainly changed my outlook on my pregnancy because you spend so much time in the hospital it removed some of that bond for me”.*

The emotional “*rollercoaster*” (*Gemma*) of the IVF treatment cycle(s) leading to the pregnancy felt to participants like an external force that was leading them up and down emotionally. This experience often left participants feeling vulnerable, anxious, distressed and depleted of resilience before embarking on pregnancy. On successful conception, participants often described feeling the need to make lifestyle changes to exert and reclaim control, as described by *Ever-Hopeful*:

*“I went for walks*,* ate all the right food. Got the right sleep*,* had a nap if I needed to. I mean*,* it was so religious…And for me*,* being in control of that was…I was doing everything that I possibly could*,* so if it did go wrong*,* I knew I had done everything I could and I couldn’t blame myself because I had done everything by the book*,* to the letter and far beyond that as well”.*

#### Subordinate theme 1.2: regaining control and empowerment through birth and feeding choices

The conception mode appeared to impact birth preferences and feeding choices. Participants’ narratives indicated that there was a desire to avoid clinical environments and a yearning for birth without medicalised intervention. *Ever-Hopeful*, who conceived using donor eggs, described her distress at requiring a caesarean birth:

*“I couldn’t do the rest of it normally*,* I wanted to have a normal birth… and now I’ve got to have a caesarean as well… You think*,* that hasn’t happened normally*,* I want something else to*”.

Contrastingly, *Jane*, who similarly conceived via donor eggs, found it difficult to contemplate vaginal delivery due to concerns regarding the risks. The baby’s health and safety were often prioritised over participants’ own wishes and preferences. A planned caesarean brought “*control” (Dee)*, certainty and containment, which was “*taken away during the IVF process*” *(Dee*).

The decision whether to breastfeed or not was often made before the baby’s arrival, and frequently embedded within family and cultural experiences. However, breastfeeding appeared to hold significant meaning for many participants (*Miriam*,* Elle*,* Tel*,* Kate*,* Sophie*) and for some their conception mode fuelled desires to feed their child “*normally”* (*Ever-Hopeful*,* Elle). Miriam* and *Ever-Hopeful* persevered with breastfeeding despite describing gruelling feeding regimes and physical pain. Participant narratives revealed that breastfeeding difficulties led to perceived failure and premature cessation was associated with “*guilt”* and feeling “*replaceable*” (*Elle).* When breastfeeding was successfully established, participants reflected on the positive effects for both infant and parent and their mutual bonding. Extended breastfeeding was discussed, surpassing participants’ expectations. For those who sought IVF for fertility challenges, initiating breastfeeding led to feeling successful and, as *Louise* reported, a sense “*that your body’s doing something right for once*”.

#### Subordinate theme 1.3: unseen and silenced

Participants contrasted in willingness to share and communicate their IVF journey with others. Stigmatisation and lacking awareness surrounding MAC often meant participants concealed their IVF journey. For some, this aspect was linked to not wanting to “*disappoint*” (*Ever-Hopeful*,* Abigail)* or feel “*pity*” (*Abigail)*, meaning participants and their partners frequently managed alone. Contrastingly, others like *Elle* were open and eager to share their IVF experience, feeling proud that their child was created with “*love and science*”. Participants described that it was difficult for conflicting emotional states to co-exist. At times this led participants to self-silence by denying and dismissing their own needs and by delaying help-seeking. *Gemma* described difficulties in accepting help postnatally, instead overcompensating and entering “*supermum*” mode.

For all participants, there was a sense that their conception mode added to feeling “*different*” at varying points throughout their antenatal or postnatal journey. Loneliness, isolation and difficulties in navigating existing relationships were noted; participants often did not vocalise concerns to healthcare professionals or their own support systems. The journey to conception also led to feeling gratitude and as a result, self-silencing. For many, there was a sense that they could not complain about difficult or challenging aspects of treatment, pregnancy or parenting for fear of being perceived as “*ungrateful*” (*Abigail*,* Gemma*,* Louise)* or “*jinxing*” (*Jane*,* Gemma*,* Louise)* their experience.

*“There was definitely an element of*,* not*,* not wanting to complain about that. Not feeling like there was space for me to complain about that*,* umm [pause] because I was so grateful. I think that’s a theme that runs through pregnancy*,* birth*,* parenthood. Trying to balance my gratitude for him* [her baby] *and that I’d been able to have him*,* with acknowledging the difficulties” (Gemma).*

In contrast, some participants reported moving from longing to profound appreciation and described savouring moments with their infant. Participants felt “*lucky*” (*Sophie*,* Abigail*,* Gemma*,* Miriam*,* Kate*, *Dee*,* Elle)*, attributing this to their journey to become parents, adding to “*patience”* (*Kate)* and feeling better equipped to deal with the challenges associated with parenting. For others, it took time to adjust and to process their baby’s safe arrival, feeling guilty for not feeling the way they thought they should feel.

*I’ve longed for this baby for so long and now I don’t even feel grateful*,* and I really felt horrendous about that. We’ve been so desperate for this baby*,* and I can’t even like*,* I didn’t feel positive about him at all….I really wanted this and now I’ve got it*,* I don’t even appreciate it…That first three months*,* I didn’t really feel excited by his arrival or anything …it felt like a shock still to be honest*,* that he was here and he was alive” (Elle).*

#### Subordinate theme 1.4: compounding losses and expanding grief

Interviews revealed the sheer extent of participants’ losses resulting from IVF which went largely unrecognised, meaning they carried unprocessed grief into their pregnancy. Participants spoke about loss and grief layers associated with MAC; a repeated, cumulative loss beginning long before successful pregnancy was achieved.

“*There is a lot of grief and loss in IVF and pregnancy. There’s the initial grief of not being able to conceive naturally. There is the grief and loss of the cycles not working initially or something going wrong or having bad news delivered to you about egg reserves*,* sperm count*,* that kind of thing. Umm*,* and then there’s sometimes the grief and loss of sometimes actually losing a pregnancy*,* a miscarriage or in my case*,* a stillbirth. So*,* there’s all these layers of loss and grief and bereavement actually whilst you’re going through the otherwise stressful journey of pregnancy” (Abigail).*

Participants reflected on loss of the hoped-for conception which often linked to intimacy, shaped by participant-perceived societal norms. The misconceptions regarding IVF in society, such as it being the “*easy*” option (*Ever-Hopeful*,* Gemma*,* Dee*) with certainty of a baby, meant individuals revealed feeling underrepresented and marginalised.

For participants who had undergone repeated IVF treatments, there was a sense of loss with each failed attempt. Some experienced miscarriage, recurrent miscarriage and, for *Abigail*, stillbirth. Their narratives indicated that these emotionally painful and traumatic experiences impacted their future pregnancy experiences.

### Superordinate theme 2: The fragility of pregnancy post-MAC - helpless and existing in a world of uncertainty

This was the theme most commonly discussed by participants. *Jane* and *Kate* likened the insurmountable feelings within the journey to pregnancy to “*climbing a mountain*”. This theme, with two subthemes, explored how participants responded to, and navigated, their pregnancy. It provides a deeper understanding of psychological responses, parental vulnerabilities, and protective measures individuals took throughout the process.

#### Subordinate theme 2.1: living in fear - doubt in the self, doubt in the process

Pregnancy after IVF was viewed with no guarantees, and an overwhelming sense of defying the odds, which contributed towards pregnancy feeling “*fragile*” (*Abigail*,* Louise)*, “*precious*” (*Ever-Hopeful*,* Elle*,* Abigail*) and the “*stakes are higher*” (*Gemma*). Participants reported shifting concerns and approached pregnancy with caution, doubt and trepidation; pregnancy loss fears were associated with anxiety, rumination and hypervigilance.

*“…just really thought*,* if we*,* if I was pregnant that the baby would die. I just really thought that I was going to have a miscarriage…I was just constantly checking for bleeding. I would be inspecting my underwear (laughter). Yeah*,* I don’t know I couldn’t accept*,* but I just found it really hard. I am not saying every moment I was a nervous wreck*,* but I would say my underlying thought was worry” (Elle).*

For some, like *Ever-Hopeful*, this was their last treatment cycle, leading to fear of losing the parental dream. Despite this, *Ever-Hopeful* reflected on her joy at the pregnancy and reported “*excitement*” as the dominant emotion, which contrasted with other interviews, characterised by disbelief and worry. Anxiety often led participants to reassurance-seeking to alleviate distress, repeatedly performing pregnancy tests and booking additional ultrasound scans. Participants reported having to see or feel the pregnancy to believe it and described “*existing*” during this time, living “*appointment to appointment*” (*Dee*,* Louise*,* Miriam).* Anxiety often accumulated before or during a scan, followed by fleeting “*euphoria*”:

*“Umm*,* but it - the euphoria- probably doesn’t even last a day*,* if that… I’m always in my head thinking*,* a scan is only as ever good as the day you’ve had it. They did help a lot. I mean*,* I couldn’t have done it without them because I just lived from scan to scan to see if it was progressing or not” (Louise).*

These fears often meant participants acted cautiously to optimise the baby’s survival. For some, this caution led to “*overthinking*” (*Sophie)* and changes to their lifestyle. One participant (*Dee*) chose to take time off work during the first trimester to reduce physical demands and stress, whilst *Frankie* perceived her employers to be overprotective and cautious when encouraging her to work from home during the Covid-19 pandemic,which contrasted with her desire to retain a sense of normality.

After multiple failed IVF cycles, *Sophie* described questioning whether or not her pregnancy was “*meant to be*”, leading to fears and reservations about her ability to “*stay pregnant*”. Those participants who sought IVF for fertility challenges often described lacking trust in their bodies’ capabilities and their fear that they could not sustain the pregnancy. They described pressure and responsibility, both adding to their apprehension and fear.

*“Errrm*,* can I do it? Am err*,* am I going to be able to do it? Am I going to be able to keep this person*,* this little thing alive? You know*,* can*,* yeah*,* can*,* can my body sustain him? Can you know*,* can I get through the next nine months without something going wrong and…you know*,* am I going to be able to keep the two of us alive kind of thing” (Frankie).*

Participant narratives revealed that the emotional complexity that accompanied pregnancy impacted their enjoyment. For many, pregnancy milestones were significant, particularly reaching 24 weeks gestation when the baby would likely survive. Throughout pregnancy anxiety remained, with a sense that participants could “*never completely relax*” (*Sophie).* Persisting fears often intensified as the birth approached, switching to stillbirth fears.

Participants reported *“disbelief*” (*Miriam*,* Abigail*,* Frankie)* and difficulties in believing the pregnancy would be successful. For many, this was viewed as a form of coping and self-protection, defending against disappointment and potential loss. Participants often reported difficulties in saying the words “*I am pregnant*” and instead opted to use scientific terminology, such as ‘embryo’, thereby creating a sense of distance and disconnect. For some, like *Gemma*, forming a childless identity helped to manage distress associated with her infertility. For others like *Tel*, difficulties believing the pregnancy were grounded in the IVF process itself:

*“And something like IVF*,* I mean we were quite lucky in that my IVF cycle worked first time*,* but that doesn’t mean that it wasn’t a traumatising experience…because your brain’s still stuck in that difficult process… you are still in so much of a mess of what’s actually happened to get there that I don’t think you can actually feel the positive result for quite a long time” (Tel).*

Participants spoke about “*thinking the worst*” (*Jane*,* Gemma*,* Elle*) to emotionally prepare themselves for any anticipated or experienced loss, meaning they were often left feeling anxious, ruminating on negative thoughts. Many reported conscious attempts to block these feelings out and described themselves as “*guarded*” (*Jane)* and emotionally detached in response.

*“…I think I was probably quite numb about it*,* like I just*,* I had got it into my head and that’s the way it’s going to be. Like*,* I wasn’t going to have this baby… Maybe it was like that…the whole process of infertility and conception and IVF had just been so overwhelming that I think my go to was “it’s not going to happen” (Elle).*

Participants reflected on their difficulties in planning and imagining a future with a baby. For many this resulted in delayed preparations, such as buying items, informing themselves regarding birth and preparing the nursery. Participants described often feeling removed or disengaged despite completing these activities.

*“My Mum and Dad did a lot of the buying because I was like “I’m not ready to do that yet”. Even getting the nursery ready*,* I didn’t go in. I didn’t go in the room. Everything was done later so my husband and my Dad did his room. Errr*,* I didn’t really. I only went in to put some towels in a cupboard*,* but that was it*,* but I would very rarely*,* and I would leave the door closed too” (Dee).*

Contrastingly, *Ever-Hopeful* started preparing for the baby’s arrival “*really early*” and reflected on her enjoyment in the process and her feelings of acceptance.

#### Subordinate theme 2.2: keeping afloat whilst riding the wave

How participants managed and coped with associated difficulties during pregnancy and the transition to parenthood was explored. Participants spoke of *“riding the wave*” of emotions *(Gemma).* Perceived social stigma and lack of acknowledgement of participants’ emotional experience led to attempts to suppress and deny their feelings. Participants described *“getting on with things*”, frequently using distractions.

Participants discussed the benefits of talking to those who understood, reflecting on the power of normalisation and shared experience. *Sophie* and *Ever-Hopeful* discussed receiving this support from their partners and support networks; others sought out belonging and connection via online communities or in-person peer support groups.

*“I was lucky to have that support group. … I think for me*,* peer support was just the most important thing in my whole journey umm*,* you know and if that could be*,* rather than it being the domain of just a random counsellor who had managed to set up this group*,* if that could be integrated into care more consistently that would be amazing” (Abigail).*

For *Elle*,* Louise*,* Gemma* and *Tel*, who did not attend a support group, there was an expressed desire for this support. Contrastingly, *Kate* did not find others’ stories helpful and was instead *“grounded*” by statistics. Participants reflected on self-empowerment through various means, such as lifestyle changes, educating themselves and/or seeking holistic care outside the NHS including acupuncture and yoga.

The ‘double-edged sword’ of monitoring fetal movements was discussed by all participants. Individuals found movements reassuring. However, fetal inactivity periods caused them acute anxiety. Participants were grateful for maternity triage services which allowed extra monitoring, helping them to contain anxiety and provide reassurance. When there was a positive, trusting relationship with the healthcare team, this was praised and reported as a protective factor.

*“It was just such a supportive environment… I think I had developed*,* a relationship with the hospital and feeling safe and knowing everything had been done to get [baby’s name] here safely and to support me and to do everything regardless of how silly my anxiety might have been or not that my anxiety was silly*,* but more the questions that I asked when I was anxious. And errm*,* the reassurance*,* it was just like*,* a weight was lifted” (Dee).*

### Superordinate theme 3:Parental function of healthcare systems - needing an anchor and a sense of safety

This theme, and its two subthemes, encapsulated varying healthcare experiences in light of IVF-assisted conception. It provided further insight into individuals’ care needs, available support and suggestions for the future.

#### Subordinate theme 3.1: Power and control in health services - a place of safety or threat

Participants described feeling “*abandoned*” (*Abigail*) and lost in *“limbo*” (*Kate*,* Abigail)* after being discharged from the fertility centres. There was a perceived “*gap*” (*Sophie)* in care before being seen by routine antenatal services.

*“…but there is a gap between 8 weeks and you being picked up and being booked by the midwife at 10–11 weeks There is a few weeks where you are in limbo land and it’s a bit weird cause you’re not really under anybody*,* only really the GP*,* so it is a bit of a weird gap…You just kinda want to get to that first midwife appointment and get to that scan and that time again*,* it just drags. It’s really*,* really long” (Sophie).*

Routine antenatal care contrasted significantly to the intense relationship with the fertility centre which involved frequent contact with familiar staff who knew their histories. *Abigail* described being “*thrust into a world of normal pregnant people*”, highlighting this sense of difference, often requiring additional support and understanding from health professionals. Other participants, such as *Tel*, experienced the transition in care positively, feeling free of IVF “*labels*”.

*“Being discharged from that side of things was actually a really nice feeling. I remember that feeling because at that point I just felt normal. Like a normal pregnant person rather than a person who was undergoing IVF treatment” (Tel).*

When transferred to routine antenatal care, little attention was given to individuals’ journeys to conception, which was often described as invalidating. When this was disclosed, participants reported feeling that health professionals had limited understanding and knowledge regarding IVF, making insensitive comments which added to their distress. Furthermore, this perceived lack of knowledge burdened participants, who were often required to take on the educator role during an emotionally vulnerable time.

*“People don’t even understand what it is [IVF]. I just felt like no-one had a clue what you are talking about. You’re kinda half educating them as you go along which you don’t want to do when you are feeling so vulnerable…” (Louise).*

Lack of understanding and awareness was also reported postnatally. *Dee* and *Miriam* reported suboptimal postnatal recognition and acknowledgement of their emotional well-being and conception history. *Miriam* discussed her distress at repeatedly being offered contraception despite declining and informing healthcare professionals of her route to parenthood and her desire to achieve pregnancy again.

The absence of ongoing contact with IVF specialists, coupled with the perceived lack of knowledge, meant that participants reported that services did not meet their needs, which then often led participants to embark on their own knowledge quest, relying on the internet and online communities to provide answers.

In addition, system disintegration was consistently reported, characterised by inconsistent responses and poor communication between healthcare professionals which caused confusion, distrust and anxiety. *Frankie* described feeling *“dictated*” to, with her own views and wishes invalidated and ignored. In contrast, as described in subordinate theme 2.2 ’*Keeping afloat whilst riding the wave’,* when a trusting relationship was developed with the healthcare teams, participants described feeling supported, held and contained, promoting feelings of safety. The healthcare teams appeared to fulfil an almost parental function, identifying and responding to their needs, providing containment both practically and emotionally.  

#### Subordinate theme 3.2: a need for specialist services

Participants reported amplified care needs based on their conception mode; for example, a need for reassurance, containment, and emotional support. Participants spoke passionately about the need for specialist services with dedicated midwives and consultants, specifically trained in fertility, MAC and perinatal loss. They valued clinicians who took the time to understand their story, acknowledged their IVF experience, and tailored care to their individual needs. Many participants expressed a need for reduced wait times, more frequent scans and appointments with consistent staff members, specifically in the first half of pregnancy when anxiety was highest. Clinicians’ interpersonal skills were frequently remarked upon. Valued qualities included interest, empathy, validation and clear, sensitive communication, being offered the opportunity to ask questions, and being given options and choices.

Despite known higher anxiety rates in individuals who become pregnant following MAC, participants reported that this did not translate into practice. This perceived lack of acknowledgement of their emotional well-being meant their psychological needs were largely neglected.

*“…no-one really sort of spoke to me about the fact that my pregnancy might be difficult after IVF in terms of anxiety*,* AT ALL…it very much felt like*,* you know*,* you’re pregnant*,* you know…enjoy it! [slight laughter]” (Miriam).*

Furthermore, psychological support was rarely available, particularly antenatally. Participants reflected on the need for continued psychological assessment and tailored psychological support beyond the IVF conception.

*“…and the offer of a counselling service. I mean there is a mental health midwife you can contact… when I was going through IVF*,* counselling was*,* it was more expected as part of it. Whereas when you were pregnant it felt like any mental health support was like an emergency kind of thing rather than*,* it being encouraged*,* and people need that” (Abigail).*

## Discussion

This study explored the lived experiences of pregnancy and transition to parenthood in individuals who conceived via IVF, identifying how they managed and coped with any associated psychological difficulties. IVF’s enduring psychological impact persisted despite diverse reasons for seeking MAC and parents were left feeling ‘psychologically wounded’ by the process leading to subsequent emotional vulnerability which was described as persisting into pregnancy and influencing birth preferences and feeding choices. The inextricable link between MAC, loss and grief was illuminated, even without history of prior perinatal or baby loss, identifying undetected and unmet parental need. The first trimester of pregnancy and the navigation of antenatal care interactions were identified as the most challenging time by participants. However, it was evident that birth, just as conception, did not erase experiences and identity as individuals who had undergone MAC, even among those without prior subfertility. Our findings identified the healthcare systems’ critical role, with potential to alleviate or perpetuate parental distress during antenatal and postnatal periods.

The literature to date has already identified the emotional complexity [11, 17, 18, 28.^29^] that accompanies pregnancy following MAC. It highlights that individuals who have conceived by MAC commonly report feeling different to other mothers [43], feelings of gratitude, self-silencing [19, 44], prioritising their baby’s health and safety [18, 45] and pregnancy loss fears [11, 46]. Fears relating to the baby’s survival were commonly linked to IVF’s impact [47]. Although these fears have been identified in the first trimester for spontaneously conceiving individuals [48], their anxiety usually decreases as pregnancy progresses [49]. For our participants, in keeping with the findings of McMahon et al. [50], these fears often persisted throughout pregnancy, transferring to stillbirth worries, even when the individual did not have a history of prior baby loss. These concerns were often not shared with others, linked to fear of judgement and pressure to appreciate their treatment [19]. 

A body of literature describes a high rate of breastfeeding initiation among IVF-conceiving individuals [51], but a shorter breastfeeding duration and higher rate of breastfeeding difficulties are frequently reported [52–55]. In this study, a determination to breastfeed and participants’ high prevalence of reported breastfeeding difficulties were identified. For our participants, breastfeeding appeared to hold significant meaning and was viewed as a way to help counteract their loss of the hoped-for conception [23, 45, 56]. Our findings described that individuals often persevered despite difficulties [45, 56] which were often internalised as a sense of failure, compounding feelings of self-perceived failure relating to the initial need for MAC.

The following observations augment our understanding of parents’ MAC experiences: the IVF journey does not stop at the successful conception, with needs evolving at each juncture. Our study revealed the sheer extent of loss and grief associated with MAC, with little opportunity to acknowledge and engage with the resultant pain and distress [57]. Healthcare systems should be attuned to potentially ongoing loss and grief [58]. 

Absence of emotional containment during this time was described as impacting psychological responses to pregnancy, often persisting after birth, taking time to process their baby’s safe arrival. While healthcare gaps are already documented [17, 19, 59], powerlessness during the MAC process left parents searching and attempting to reclaim control in pregnancy. We identified that personalised healthcare promoted empowerment and a sense of safety. Together, this highlights healthcare systems’ significance and need for practical and emotional containment, when individuals are vulnerable psychologically. When parents enter pregnancy vulnerable, their attachment systems are likely to become activated [60, 61]. Building trusting relationships with consistent staff is then fundamental in maintaining relational safety.

Exploring how individuals managed and coped with the difficulties that arose in pregnancy and parenting following MAC (specifically including those using MAC for non-subfertility indications) has been poorly described in the literature to date. Individuals psychologically defend themselves using self-protection, with reliance on avoidance and suppression linked to poorer emotional well-being [62]. Thwarted belonginess [63, 64] suggests that distress and psychological pain are occurring when the human need for connection and belonging is unmet, which illuminates the power of shared experience. In-person support groups were deemed invaluable. Being able to reflect with people who truly understood helped normalise our participants’ experiences, adding to their feelings of acceptance and belonging.

### Clinical and research implications

These 12 participants’ narratives indicated that the psychological needs of individuals who become pregnant after MAC were largely neglected, but were similar even outside the context of subfertility. Holistic antenatal and postnatal care, including psychological awareness, screening and support, is required to help individuals process and integrate their experiences associated with MAC, as recommended in the Royal College of Nursing ‘Transition from Fertility to Maternity Care’ guidance [65]. In line with the Department of Health ‘The 1001 Critical Days’ manifesto [32], the period from conception to age two is critical for baby’s brain development, laying the foundations for the child’s future cognitive, social, and emotional well-being [66, 67]. Therefore, psychological care in pregnancy would benefit the prospective parent and the developing child. Embedding health clinical psychologists (or psychology services) within maternity services would normalise and reinforce this care. Facilitated peer support groups and greater consistency of carers, if possible, are also recommended.

### Strengths and limitations

Historically, research into psychological well-being and adaptation to pregnancy and parenting after MAC has been embedded within an infertility context. A study strength was including individuals who sought IVF for non-subfertility reasons, demonstrating the shared psychological impact of MAC itself. Including multiple perspectives allowed a more comprehensive view. However, caution is still required when relating these findings to MAC as three-quarters of participants sought IVF for subfertility, and it is possible that their narratives were a consequence of subfertility, MAC or both. Nevertheless, this study was designed to focus on the MAC pregnancy experience and not subfertility, reflected in our recruitment and interview procedures. Despite participant diversity in sexual orientation, gender, family formation, geographical location as well as prior IVF experiences, the data showed that experiences were quite similar across participants, reflecting a shared experience. However, future studies may focus specifically on diversity within participants’ needs and experiences.

All participants self-referred into the study suggesting greater motivation to share their story, risking selection bias. Additionally, participants’ pregnancy, birth and parenthood transition experiences were in the UK’s Covid-19 pandemic context, which is likely to have increased anxiety and reduced access to coping resources, influencing experiences during IVF treatment, pregnancy and transition to parenthood. No data were collected on whether the IVF treatment was publicly or privately funded, which could have influenced the experience of pregnancy after treatment. However, all participants had their IVF treatment in the UK. Additionally, all participants identified as White British, were highly educated and had one living child, meaning the voices of parents with previous children and from different social backgrounds were not captured. Although Black and ethnic minority background individuals’ recruitment was encouraged through inclusive study advertisements; no individuals from these backgrounds expressed interest. This is a general literature weakness and a priority for future research. Finally, this study was conducted in the UK, with universal access to publicly funded obstetric care, meaning the results may not be transferable to other contexts.

## Conclusions

This study exploration of the lived experiences of individuals who conceived via MAC outlines a continuation of the IVF journey throughout pregnancy, birth and transition to parenthood. It highlights IVF treatment’s persistent psychological impact, whereby individuals enter pregnancy emotionally vulnerable and resilience-depleted. Participants expressed enhanced psychological care needs throughout the perinatal period. The expectant parents’ psychological vulnerability after MAC needs to be addressed throughout pregnancy and the perinatal period by maternity healthcare providers.

## Electronic supplementary material

Below is the link to the electronic supplementary material.

Supplementary Material 1

## Data Availability

The datasets generated and/or analysed during this study are not publicly available due to lack of consent from participants to make whole interview transcripts available. But upon reasonable request they can be made available from the first and/or corresponding author.
