# Supplementary material for: Life after medicalised conception: an interpretative phenomenological analysis study exploring the experiences of pregnancy and transition to parenthood
Source: BMC Pregnancy Childbirth. 2025 Feb 11;25:146. doi: 10.1186/s12884-025-07226-7 (PMC11817777; doi:10.1186/s12884-025-07226-7)
Supplement: Supplementary file 1 — Supplementary Material 1 [file 12884_2025_7226_MOESM1_ESM.docx]

**Supplementary Table: Five sections of the interview and example questions**

| **Five sections to this interview** | **Opening question** | **Prompts** |
| --- | --- | --- |
| Early pregnancy experience | Please can you tell me about your experience at the start of pregnancy? i.e. after you found out you were pregnant? | Can you tell me a little more please  Please explain further…  Any examples?  Why do you think that happened?  What was that like for you? |
| Late pregnancy experience | Please can you tell me about your experience towards the end of pregnancy i.e. preparing for the birth? | Can you tell me a little more, please explain further, any examples?  Why do you think that happened?  What was that like for you? |
| Childbirth | Can you tell me about your experience of childbirth? | Can you tell me a little more…  Please explain further  Any examples  Why do you think that happened?  What was that like for you? |
| Early parenting experiences | Can you tell me about your parenting experience so far? | Can you tell me a little more?  Please explain further  Why do you think that happened?  What was that like for you? |
| closing statement | Is there anything that we haven’t covered already that you would like to talk about before we finish? |  |
